# Supplementary material for: A Critical View of the Application of the APEX Software (Aqueous Photochemistry of Environmentally-Occurring Xenobiotics) to Predict Photoreaction Kinetics in Surface Freshwaters
Source: Molecules. 2019 Dec 18;25(1):9. doi: 10.3390/molecules25010009 (PMC7017383; doi:10.3390/molecules25010009)
Supplement: Supplementary file 1 [file molecules-25-00009-s001.zip › 3.2.4_gcc-4.4.0.zip!/3.2.4_gcc-4.4.0/3.2.4_gcc-4.4.0/doc/octave/html/interpreter/How-to-Contribute.html]

How to Contribute - Untitled


Next: General Guidelines,
Up: Contributing Guidelines


---

### D.1 How to Contribute

The mailing list for Octave development discussion and sending contributions is
maintainers@octave.org. This concerns the development of Octave core,
i.e., code that goes to Octave directly. You may consider developing and
publishing a package instead; a great place for this is the allied Octave-Forge
project (http://octave.sf.net). Note that the Octave project is
inherently more conservative and follows narrower rules.

The preferable form of contribution is creating a Mercurial changeset and
sending it via e-mail to the octave-maintainers mailing list. Mercurial is the
source code management system currently used to develop Octave. Other forms of
contributions (e.g., simple diff patches) are also acceptable, but they slow
down the review process. If you want to make more contributions, you should
really get familiar with Mercurial. A good place to start is
http://www.selenic.com/mercurial/wiki/index.cgi/Tutorial. There you will
also find help how to install Mercurial.

A simple contribution sequence could look like this:

```
     hg clone http://www.octave.org/hg/octave
                                  # make a local copy of the octave
                                  # source repository
     cd octave
     # change some sources...
     hg commit -m "make Octave the coolest software ever"
                                  # commit the changeset into your
                                  # local repository
     hg export -o ../cool.diff tip
                                  # export the changeset to a diff
                                  # file
     # send ../cool.diff via email
```

You may want to get familiar with Mercurial queues to manage your changesets.
Here is a slightly less simple example using Mercurial queues, where you work
on two unrelated changesets in parallel and update one of the changesets after
discussion in the maintainers mailing list:

```
     hg qnew nasty_bug            # create a new patch
     # change sources...
     hg qref                      # save the changes into the patch
     # change even more...
     hg qref -m "solution to nasty bug!"
                                  # save again with commit message
     hg export -o ../nasty.diff tip
                                  # export the patch
     # send ../nasty.diff via email
     hg qpop                      # undo the application of the patch
                                  # and remove the changes from the
                                  # source tree
     hg qnew doc_improvements     # create an unrelated patch
     # change doc sources...
     hg qref -m "could not find myfav.m in the doc"
                                  # save the changes into the patch
     hg export -o ../doc.diff tip
                                  # export the second patch
     # send ../doc.diff tip via email
     hg qpop
     # discussion in the maintainers mailing list ...
     hg qpush nasty_bug           # apply the patch again
     # change sources yet again ...
     hg qref
     hg export -o ../nasty2.diff tip
     # send ../nasty2.diff via email
```
